# Treatment and preliminary outcomes of 150 acute care patients with COVID-19 in a rural health system in the Dakotas

**DOI:** 10.1017/S0950268820001351

**Published:** 2020-06-22

**Authors:** M. O. Enzmann, M. P. Erickson, C. J. Grindeland, S. M. C. Lopez, S. E. Hoover, D. D. Leedahl

**Affiliations:** 1 Pharmacy Services, Sanford Medical Center, Fargo, ND, USA; 2 Department of Infectious Diseases, Sanford USD Medical Center, Sioux Falls, SD, USA

**Keywords:** Coronavirus, COVID-19, SARS-CoV-2

## Abstract

The majority of available US-published reports present populations with community spread in urban areas. The objective of this report is to describe a rural healthcare system's utilisation of therapeutic options available to treat Coronavirus Disease 2019 (COVID-19) and subsequent patient outcomes. A total of 150 patients were treated for COVID-19 at three hospitals in the Dakotas from 21 March 2020 to 30 April 2020. The most common pharmacological treatment regimens administered were zinc, hydroxychloroquine plus azithromycin and convalescent plasma. Adjunctive treatments included therapeutic anticoagulation, tocilizumab and corticosteroids. As of 1 June 2020, 127 patients have survived to hospital discharge, 12 patients remain hospitalised and 11 patients have expired. The efficacy of hydroxychloroquine and azithromycin use has yet to be determined but was not without risks of corrected QT interval prolongation and arrhythmias in our cohort. We did not appreciate any adverse effects that appeared related to tocilizumab or convalescent plasma administration in those patient subsets. These findings may provide insight into disease severity and treatment options in the rural setting with limited resources to participate in clinical trials and encourage larger comparative studies evaluating treatment efficacy.

Coronavirus disease 2019 (COVID-19) and its causal pathogen, severe acute respiratory syndrome coronavirus-2 (SARS-CoV-2), have been classified by the World Health Organization as a pandemic with over 6 000 000 cases confirmed globally [1, 2]. The majority of available United States (US)-published reports present populations with community spread in urban areas [3, 4]. In this letter, we describe the characteristics, pharmacologic treatment and preliminary outcomes of 150 acute care patients with COVID-19 within three hospitals of Sanford Health, an integrated healthcare system in the upper Midwest. North Dakota and South Dakota represent rural areas of the USA. The median incomes of North Dakota and South Dakota in 2018 were $ 63 837 and $ 56 274, respectively. As of April 2020, North Dakota has a civilian labour force of 407 100 people with an unemployment rate of 8.5%, while South Dakota has a civilian labour force of 470 100 people with an unemployment rate of 10.2% [5]. Infectious Diseases Society of America COVID-19 Treatment Guidelines published on 11 April 2020 only recommend treatment of COVID-19 with pharmacological agents in the context of a clinical trial; however, rural health systems may not always have the capabilities and resources necessary to rapidly join open clinical trials [6]. Thus, we feel it is important to outline how a rural healthcare system has deployed the therapeutic options available for COVID-19.

In our study, patients with confirmed SARS-CoV-2 infection by positive nasopharyngeal polymerase chain reaction test seen in the emergency department or admitted to one of three Sanford Health hospitals between 21 March 2020 and 30 April 2020 were included. Participants were enrolled in an IRB-approved registry-based cohort with a waiver of informed consent. The data presented were collected through review of secure, confidential electronic health records that were accessed with permission from the IRB, but are not publicly available.

A total of 150 patients were included; 56.7% were male (*n* = 85), with a median age of 56 years (range: 1 month–95 years), and 95 (63.3%) were Caucasian. Demographic and clinical characteristics are described in Table 1. The most common comorbidities observed were cardiovascular (CV) disease and diabetes mellitus. Fourteen patients were never admitted for inpatient treatment, but rather discharged home after their emergency department encounter. Abnormal chest radiograph findings were observed in 89 patients and common admission symptoms included cough, shortness of breath and fever.

**Table 1.** Baseline characteristics, treatment and preliminary outcomes of 150 hospitalised patients with COVID-19

Demographics	Value	
Age (in years), median (range)	56 (1 month–95 years)	
Sex, number (%)		
Male	85 (56.7)	
Female	65 (43.3)	
Race, number (%)		
Caucasian	95 (63.3)	
American Indian	26 (17.3)	
African-American	18 (12.0)	
Asian	5 (3.3)	
Hispanic/Latino	3 (2.0)	
Pacific Islander	2 (1.3)	
Not documented	2 (1.3)	
Baseline characteristics	*Number (%) of patients*	
Asthma	*27 (18.0)*	
Chronic obstructive lung disease	*16 (10.7)*	
Sleep apnoea	*22 (14.7)*	
Congestive heart failure	*16 (10.7)*	
Cardiovascular disease^a^	*77 (51.3)*	
Diabetes mellitus	*43 (28.7)*	
Rheumatologic disease	*7 (4.7)*	
Chronic kidney disease	*23 (15.3)*	
End-stage renal disease	*8 (5.3)*	
Liver cirrhosis	*4 (2.7)*	
History of solid organ transplant	*0*	
Current smoker	*24 (16.0)*	
Immunosuppression^b^	*7 (4.7)*	
Admission symptoms		
Cough	93 (62.0)	
Shortness of breath	95 (63.3)	
Fever^c^	92 (61.3)	
Admission chest radiograph findings^d^		
Airspace opacities	27 (18.0)	
Atelectasis	21 (14.0)	
Groundglass opacities	9 (6.0)	
Focal consolidation	4 (2.7)	
Pleural effusion	28 (18.7)	
Clear	24 (16.0)	
Laboratory test	Value median (range)	Reference range
Admission laboratory values		
White blood cell (count/μl)	2.5 (1.6–20.3)	4.0–11.0
Absolute lymphocyte count (K/μl)	1.0 (0.2–4.8)	0.8–4.1
Haemoglobin (g/dl)	13.0 (5.8–17.1)	13.5–17.5
Platelets ( × 10^3^ μl)	195 (56–622)	140–400
Ferritin (ng/ml)	385 (2–14 801)	21–275
Glucose (mg/dl)	112 (57–614)	70–99
Sodium (mEq/l)	137 (120–149)	136–145
Creatinine (mg/dl)	0.93 (0.47–12.1)	0.80–1.30
Alkaline phosphatase (U/l)	78 (22–336)	30–150
Aspartate aminotransferase (U/l)	41 (10–724)	0–355
Alanine aminotransferase (U/l)	27 (7–477)	0–55
Creatinine kinase (U/l)	96 (25–1215)	30–200
Venous lactate (mmol/l)	1.2 (0.5–5.3)	0.5–2.2
D-dimer (μg/ml)	0.8 (<0.27–8.0)	≤0.49
CRP (mg/l)	57.5 (0.9–299)	0.00–1.00
Procalcitonin (ng/ml)	0.13 (0.02–2.64)	<0.07
Treatment or outcome	Number (%) of patients	
Pharmacologic agent		
Hydroxychloroquine + azithromycin^e^	66 (44.0)	
Hydroxychloroquine monotherapy	9 (6.0)	
Lopinavir/ritonavir	3 (2.0)	
Convalescent plasma	16 (10.7)	
Tocilizumab	12 (8.0)	
IL-6 value, median (range)	40.1 (1.0 to >400)	
Zinc	93 (62.0)	
Ascorbic acid	13 (8.7)	
Angiotensin receptor blocker^f^	6 (4.0)	
Corticosteroid	39 (26.0)	
Non-steroidal anti-inflammatory	12 (8.0)	
Therapeutic anticoagulant	52 (34.7)	
Intravenous immune globulin	1 (0.7)	
Potential adverse effects		
Retinopathy	0	
Arrhythmia confirmed by ECG after H + A initiation^g^	14 (18.7)^h^	
QTc > 500 ms confirmed by ECG after H + A initiation	15 (20.0)^h^	
Outcomes		
ICU admission	38 (25.3)	
O_2_ requirement	89 (59.3)	
Non-invasive positive pressure ventilation	23 (15.3)	
Invasive mechanical ventilation	28 (18.7)	
Acute respiratory distress syndrome^i^		
None	110 (73.3)	
Mild	6 (4.0)	
Moderate	21 (14.0)	
Severe	13 (8.7)	
Vasopressor use	28 (18.7)	
AKI^j^	31 (20.7)	
Length of stay (days)^k^		
Mean	8.1	
Median (range)	5 (1–64)	
In-hospital mortality	11 (7.3)	

CRP, C-reactive protein; AKI, acute kidney injury; ECG, electrocardiogram; ICU, intensive care unit; IL-6, interleukin-6; O_2_, oxygen; QTc, corrected QT interval.

a Defined as history of coronary artery disease, myocardial infarction, valvular disease, heart transplant or hypertension.

b Defined as chemotherapy use, outpatient prescription of greater than 10 mg/day of prednisone for ≥3-week duration or use of non-steroidal immunosuppressive agents for transplant or for autoimmune disease.

c Defined as temperature greater than 100.4°F.

d According to radiologist physician interpretation in medical record.

e 52 out of 66 patients who received H + A received hydroxychloroquine 400 mg twice daily for 2 doses, followed by 200 mg twice daily as well as azithromycin 500 mg on day 1, followed by 250 mg daily thereafter.

f All patients had previously been taking an angiotensin receptor blocker prior to hospital admission and was continued for blood pressure control.

g Hydroxychloroquine and azithromycin.

h Percentage of the 75 patients who received hydroxychloroquine.

i Based on Berlin Criteria.

j Based on criteria defined by Kidney Disease Improving Global Outcomes.

k Not including patients still hospitalised in a Sanford facility as of 1 June 2020.

Pharmacological treatments prescribed for the treatment of COVID-19 and its related sequelae are summarised in Table 1. Patients were selected to receive different pharmacological treatments based on physician discretion with the support of an internal COVID-19 treatment guidance document that was continuously updated as new peer-reviewed literature became available. Risks and benefits of each treatment were explained within this document as well as guidance on which subsets of patients may benefit from certain treatments over others. The median length of time after admission that patients received pharmacological treatment was less than 1 day. At least a 5-day course of hydroxychloroquine and azithromycin was prescribed to 66 out of 150 COVID-19 patients. Sixteen patients received convalescent plasma. Of note, tocilizumab was administered to 12 patients with critical illness and elevated interleukin-6 (IL-6) levels (>1.8 pg/ml) and/or elevations in other inflammatory markers. Five patients received two doses. Other therapies included zinc, ascorbic acid, lopinavir/ritonavir, non-steroidal anti-inflammatory agents, therapeutic anticoagulation and corticosteroids. When critically ill patients appeared refractory to other therapies or supportive care, convalescent plasma was pursued. No patient in our cohort received remdesivir, as it was not yet available to our hospitals outside of the manufacturer's compassionate use programme for pregnant or paediatric patients.

Preliminary outcomes are also described in Table 1. As of 1 June 2020, acute respiratory distress syndrome (ARDS) occurred in 40 out of 150 patients, with 28 of 40 ARDS patients requiring mechanical ventilation. Vasopressor support was administered for 28 patients, and acute kidney injury diagnosed in 31 patients. A total of 127 patients have survived to hospital or emergency department discharge to date. All 14 patients who were never admitted for inpatient treatment remain alive as of 1 June 2020. Of those 14 patients, only three received pharmacologic treatment specifically for COVID-19 diagnosis, which was in the form of a 5-day course of azithromycin. Follow-up was completed in the form of appointments with a provider (in-person or via telephone) after emergency department discharge. Four of the 14 patients not admitted for inpatient treatment do not have follow-up appointments scheduled as of 1 June 2020. Of the 10 patients who had follow-up appointments, the mean follow-up period was 14 days after their emergency department visit. As of 1 June 2020, 12 patients remain alive and hospitalised (eight intensive care unit, three general ward) and 11 patients did not survive to discharge. Of the 11 patients who did not survive to discharge, seven received hydroxychloroquine and azithromycin. Of note, one patient was transferred to a higher level of care for extracorporeal membrane oxygenation. The average length of stay (not including patients still hospitalised in a Sanford facility as of 1 June 2020) in this cohort was approximately 8 days and the median length of stay was 5 days (range 1–64).

This report is the first to describe the treatment of acute care patients within a rural healthcare system in the USA, which includes general ward and critically ill patients. Hydroxychloroquine and azithromycin appeared to be a common treatment strategy in our cohort, but its impact on disease progression or survival is unclear, and 11 deaths have occurred in this cohort as of 1 June 2020 (the first confirmed COVID-positive patient was admitted to our hospital system on 21 March 2020). All patients but one who received tocilizumab had an IL-6 serum level above the upper limit of normal of 1.8 pg/ml. The patient who received tocilizumab without an elevated IL-6 serum level had elevations in other inflammatory markers (ferritin, C-reactive protein and D-dimer). As with other therapies, the impact of convalescent plasma on survival is not yet clear.

This report potentially signals a lower hospital mortality rate than the current national average. However, it is unknown how the COVID-19 mortality rate in the Dakotas will change as the projected surge in COVID-19 approaches [2]. The efficacy of hydroxychloroquine and azithromycin use, with or without tocilizumab, has yet to be clarified but was not without risks of corrected QT interval (QTc) prolongation and arrhythmias in our cohort. Of the 75 patients who received hydroxychloroquine, 14 (18.7%) developed an arrhythmia and 15 (20.0%) developed a QTc > 500 ms after initiation of therapy. Baseline EKGs did not reveal the presence of arrhythmias or prolonged QTc in these patients prior to the initiation of hydroxychloroquine and azithromycin therapy. Additionally, 10 of 14 (71%) patients with arrhythmia development had underlying CV disease, while 10 of 15 (67%) patients with prolonged QTc > 500 ms had underlying CV disease. Of the nine patients that received hydroxychloroquine without azithromycin, none developed an arrhythmia or QTc > 500 ms after therapy initiation. The incidence of arrhythmias after initiation of hydroxychloroquine and azithromycin in our cohort (18.7%) appears to be higher than the incidence of arrhythmias after initiation of hydroxychloroquine and a macrolide antibiotic (8.1%) in the largest multinational registry analysis (over 96 000 patients) of hospitalised COVID-19 patients to date, although caution should be exercised in interpreting this comparison given the small size of our cohort [7]. Diarrhoea occurred in 19 (12.7%) patients, constipation developed in 9 (6.0%) patients and nausea and/or vomiting occurred in 22 (14.7%) patients. Of note, it is possible that these gastrointestinal occurrences could be attributed to the SARS-CoV-2 virus itself rather than side effects of medications. Furthermore, we did not appreciate any adverse effects that appeared related to tocilizumab or convalescent plasma administration in those patient subsets.

Similar to other recent US reports, our data are limited by a small but important sample and the use of preliminary outcomes. However, our findings may provide insight into the severity of the disease across a rural acute care cohort and the agents utilised for treatment when rapid clinical trial access may not be feasible. We recognise that the unavailability of symptom duration prior to inpatient admission is a limitation of our report, which may have helped to better characterise disease progression and duration. Statistical comparisons between treatment groups were also not pursued given the non-randomised patient selection and small sample size. The use of medications in rural hospitals to treat COVID-19 infection has yet to be clarified, but is not without risks, especially with regards to the combination of hydroxychloroquine and azithromycin in patients with CV comorbidities. Larger trials with randomisation methods examining the efficacy and safety of pharmacologic therapies to treat COVID-19 are needed urgently.

## Data Availability

The data that support the findings of this study are available from Sanford Research. Restrictions apply to the availability of these data, which were used under license for this study. Data are available from the authors with the permission of Sanford Research and the Sanford Institutional Review Board.

## Appendix A

See Table A1.

**Table A1.** Baseline characteristics and preliminary outcomes by treatment group^a^

	Number (%) of patients			
	Hydroxychloroquine + azithromycin cohort (*n* = 66)^b^	Tocilizumab cohort (*n* = 12)	Convalescent plasma cohort (*n* = 16)	All hospitalised patients (*n* = 136)
Asthma	13 (19.7)	3 (25.0)	4 (25)	25 (18.4)
Chronic obstructive lung disease	8 (12.1)	2 (16.7)	3 (18.8)	16 (11.8)
Sleep apnoea	12 (18.1)	3 (25.0)	3 (18.8)	21 (15.4)
Congestive heart failure	7 (10.6)	1 (8.3)	1 (6.3)	16 (11.8)
Cardiovascular disease^c^	40 (60.6)	9 (75.0)	12 (75)	70 (51.5)
Diabetes mellitus	26 (39.4)	7 (58.3)	7 (43.8)	43 (31.6)
Rheumatologic disease	2 (3.0)	1 (8.3)	0	6 (4.4)
Chronic kidney disease	16 (24.2)	4 (33.3)	3 (18.8)	22 (16.2)
End-stage renal disease	6 (9.1)	2 (16.7)	0	8 (5.9)
Liver cirrhosis	2 (3.0)	0	0	4 (2.9)
History of solid organ transplant	0	0	0	0
Current smoker	10 (15.2)	1 (8.3)	3 (18.8)	21 (15.4)
Immunosuppression^d^	4 (6.1)	0	0	6 (4.4)
Outcomes	Number (%) of patients			
ICU admission	29 (43.9)	12 (100)	15 (93.8)	38 (27.9)
O_2_ requirement	53 (80.3)	12 (100)	14 (87.5)	89 (65.4)
Non-invasive positive pressure ventilation	16 (24.2)	9 (75.0)	10 (62.5)	23 (16.9)
Invasive mechanical ventilation	25 (37.9)	10 (83.3)	11 (68.8)	28 (20.6)
Acute respiratory distress syndrome^e^				
None	35 (53.0)	0	0	96 (70.6)
Mild	5 (7.6)	1 (8.3)	2 (12.5)	6 (4.4)
Moderate	17 (25.8)	5 (41.7)	10 (62.5)	21 (15.4)
Severe	9 (13.6)	6 (50.0)	4 (25.0)	13 (9.6)
Vasopressor use	24 (36.4)	12 (100)	13 (81.3)	28 (20.6)
AKI^f^	20 (30.3)	7 (58.3)	7 (43.8)	31 (22.8)
Length of stay (days)				
Mean	8.0	26.4	20.6	8.1
Median (range)	5 (1–64)	22 (10–64)	19 (8–36)	5 (1–64)
In-hospital mortality	7 (10.6)	3 (25.0)	5 (31.3)	11 (8.1)

AKI, acute kidney injury; ICU, intensive care unit; O_2_, oxygen.

a Treatment cohorts are not mutually exclusive.

b 52 out of 66 patients received hydroxychloroquine 400 mg twice daily for 2 doses, followed by 200 mg twice daily as well as azithromycin 500 mg on day 1, followed by 250 mg daily thereafter.

c Defined as history of coronary artery disease, myocardial infarction, valvular disease, heart transplant or hypertension.

d Defined as chemotherapy use, outpatient prescription of greater than 10 mg/day of prednisone for ≥3-week duration or use of non-steroidal immunosuppressive agents for transplant or for autoimmune disease.

e Based on Berlin Criteria.

f Based on criteria defined by Kidney Disease Improving Global Outcomes.
